# mTORC1-mediated polarization of M1 macrophages and their accumulation in the liver correlate with immunopathology in fatal ehrlichiosis

**DOI:** 10.1038/s41598-019-50320-y

**Published:** 2019-10-01

**Authors:** Mohamed Haloul, Edson R. A. Oliveira, Muhamuda Kader, Jakob Z. Wells, Tyler R. Tominello, Abdeljabar El Andaloussi, Cecelia C. Yates, Nahed Ismail

**Affiliations:** 1 Department of Pathology, College of Medicine, University of Illinois at Chicago, Chicago, IL USA; 2 Children’s Cancer Hospital Egypt, 57357 Cairo, Egypt; 3 Department of Pathology, School of Medicine, University of Pittsburgh, Pittsburgh, PA USA; 4 Nursing School, University of Pittsburgh, Pittsburgh, PA USA

**Keywords:** Pathogens, Bacterial infection

## Abstract

A polarized macrophage response into inflammatory (M1) or regenerative/anti-inflammatory (M2) phenotypes is critical in host response to multiple intracellular bacterial infections. *Ehrlichia* is an obligate Gram-negative intracellular bacterium that causes human monocytic ehrlichiosis (HME): a febrile illness that may progress to fatal sepsis with multi-organ failure. We have shown that liver injury and *Ehrlichia*-induced sepsis occur due to dysregulated inflammation. Here, we investigated the contribution of macrophages to *Ehrlichia*-induced sepsis using murine models of mild and fatal ehrlichiosis. Lethally-infected mice showed accumulation of M1 macrophages (iNOS-positive) in the liver. In contrast, non-lethally infected mice showed polarization of M2 macrophages and their accumulation in peritoneum, but not in the liver. Predominance of M1 macrophages in lethally-infected mice was associated with expansion of IL-17-producing T, NK, and NKT cells. Consistent with the *in vivo data*, infection of bone marrow-derived macrophages (BMM) with lethal *Ehrlichia* polarized M0 macrophages into M1 phenotype under an mTORC1-dependent manner, while infection with non-lethal *Ehrlichia* polarized these cells into M2 types. This work highlights that mTORC1-mediated polarization of macrophages towards M1 phenotype may contribute to induction of pathogenic immune responses during fatal ehrlichiosis. Targeting mTORC1 pathway may provide a novel aproach for treatment of HME.

## Introduction

Human monocytic ehrlichiosis (HME) is an emerging life-threatening tick-borne disease caused by the obligate intracellular bacterium *Ehrlichia chaffeensis*. HME is often presented as a nonspecific syndrome of fever, headache, malaise, and myalgias. However, in some cases the disease can evolve to a severe form which is commonly marked by acute liver inflammation followed by multi-organ dysfunction and toxic shock-like syndrome^1,2^. Protective immunity against *Ehrlichia* is mediated by IFN-*γ*-producing CD4 Th1 cells and NKT cells. However, these cells undergo suppression and then apoptosis during fatal ehrlichiosis, which results in impaired protective immunity and defective bacterial clearance. Additionally, fatal ehrlichiosis is also attributed to development of an excessive inflammation and expansion of pathogenic NK cells, neutrophils, and CD8 T cells causing tissue injury^3–6^. Macrophages are the major target cells for *Ehrlichia*. Macrophages are important innate cells involved in homeostatic and pathological processes during infections, as they link innate and adaptive responses^7–9^. These cells are described as either resident cells, which are generally associated with maintenance of tissue integrity^10–12^, or monocyte-derived, that under specific environmental conditions play a major role in modulating inflammation^13–15^. Distinct sets of environmental stimuli guide macrophages to particular phenotype programs known as macrophage polarization status^16,17^. Polarized macrophages are classically grouped into two types: (i) the M1-type, which is formed by typical inflammatory cells classically generated upon induction by microbial products and pro-inflammatory cytokines, and (ii) the M2-type, that is ultimately associated with the promotion of tissue remodeling and repair^18–20^. M1 macrophages are characterized by enhanced expression of MHC class II, high production of pro-inflammatory cytokines, such as TNF-*α*, IL-12 and IL-23, increased expression of inducible nitric oxide synthase (iNOS), downregulation of surface mannose receptor (MRC1/CD206)^18,21,22^, and were recently associated with the induction of Th17 response^23^. On the other hand, the M2-macrophage group is marked by enhanced expression of CD206, arginase-1, increased production of TGF-*β* and IL-10, enhanced autophagic activity, among other characteristics^15,24–28^. Predominance of specific macrophage-type functions could either lead to protective roles by increasing homeostatic activity or enhanced pathogenesis though exaggeration of harmful inflammatory signals. The role of macrophage heterogeneity in host resistance and susceptibility to fatal ehrlichiosis is not understood. In this study, we investigated the macrophage heterogeneity in murine models of mild and fatal ehrlichiosis caused by infection of C57BL/6 mice with mildly virulent *Ehrlichia muris* or highly virulent *Ixodes ovatus ehrlichia* (IOE). These models recapitulate clinical and pathologic findings in patients with ehrlichiosis. Our data demonstrate accumulation of iNOS-producing monocyte-derived macrophages in the liver of IOE-, but not in *E. muris*-infected animals. In contrast, M2 macrophages were more abundant in the peritoneum of *E. muris*-infected mice. Expansion of M1 macrophages in IOE-infected mice was associated with induction of IL-17 production by multiple cell subsets including T, NK and NKT cells. Additionally, we show that IOE-induced mTORC1 activation may account for macrophage polarization into M1 phenotype during fatal *Ehrlichia* infection.

## Results

### M1-macrophage type accumulates in the liver of C57BL/6 mice infected with fatal *Ehrlichia*

Consistent with our previous studies^29,30^, infection of WT C57BL/6 mice with a high lethal dose of virulent IOE (10^4^ organisms/mouse) is associated with high bacterial burden in the liver on day 7 post infection (p.i.), when compared to mice infected with mildly virulent *E. muris* (see Supplementary Fig. S1). IOE-infected mice developed severe liver damage as marked by the presence of multiple foci of apoptotic/necrotic hepatocytes and cells lining liver sinusoids (e.g. macrophages and endothelial cells), Kupffer cells, and hepatocytes on day 7 p.i., compared to uninfected mice. In contrast, liver of *E. muris*-infected mice showed minimal pathology at this same time point (see Supplementary Fig. S1). As we have shown before^4^, IOE infection resulted in 100% mortality at 8–10 days p.i., while all mice infected with *E. muris* survived till day end of the experiments (day 30 p.i.). Our previous studies demonstrated spatial and temporal changes in immune responses mediated by NK cells, neutrophils, and CD8 T cells, where these cells migrate to the liver and expand within the inflammatory hepatic microenvironment during the course of IOE infection^4,31^. We hypothesized that the changes in innate and adaptive immunity following IOE infection could be due to differences in macrophage polarization and function.

**Figure 1:**
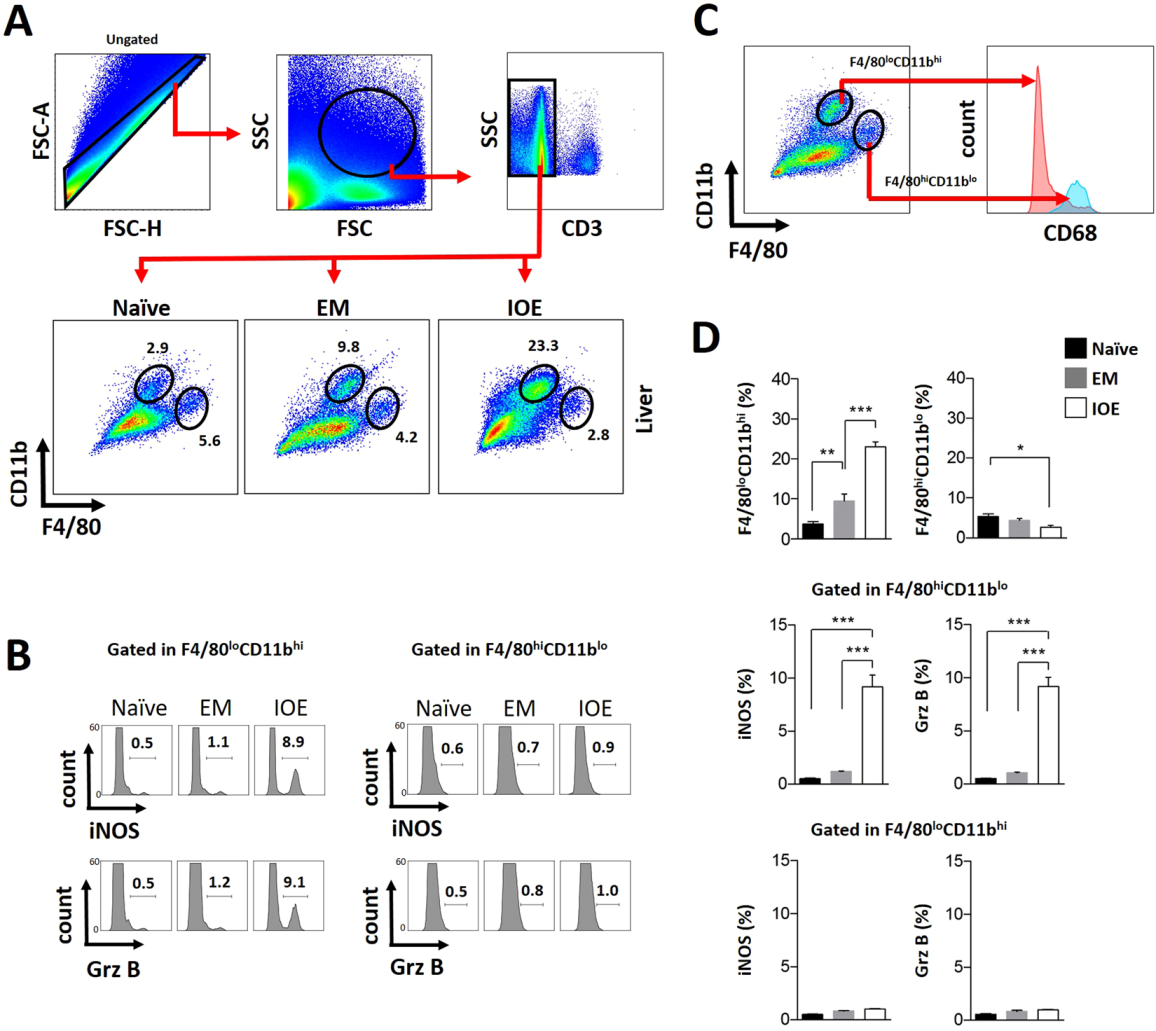
Flow cytometry analysis of liver samples from *Ehrlichia*-infected mice. **(A**) Flow cytometry gate strategy for the analysis of liver samples. Ungated events were initially plotted as FSC-A × FSC-H for exclusion of cell aggregates located far from the main diagonal. Considered cells were then morphologically represented in SSC × FSC density plots where a region containing mainly CD11b^+^ and F4/80^+^ cells (as confirmed by back gating) was defined. From these considered events, CD3-positive cells were excluded and the resultant events were plotted as CD11b × F4/80 for the analysis of infiltrating macrophages/monocytes defined as F4/80^lo^CD11b^hi^ cells, and the Kupffer cells, considered as F4/80^hi^CD11b^lo^ cells. Values represent frequencies in the assigned regions. (**B)** Each of the considered macrophage populations was also addressed for the intracellular expression of iNOS and granzyme B (Grz B) and represented as flow histograms. Values represent frequencies in the assigned regions. (**C**) Differential expression of CD68 between non-resident macrophages and Kupffer cells. Flow cytometry dot plot representative of an *E. muris*-infected animal exhibiting the considered regions for Kupffer cells (F4/80^hi^CD11b^lo^ cells) and non-resident or infiltrating macrophages/monocytes (F4/80^lo^CD11b^hi^ cells). Each of these regions were checked for the expression of CD68 and as expected Kupffer cells are positive and non-resident cells are negative as shown by the flow histogram overlay. This observed pattern of CD68 expression held for every analyzed groups, naive, *E. muris* (EM) and *Ixodes Ovatus Ehrlichia* (IOE). Events in the blue peak are gated in F4/80^hi^CD11b^lo^ region and events from the red peak are gated on the F4/80^lo^CD11b^hi^ region. (**D**) Quantification of the analyzed cell sub-populations in the different studied groups. Values are expressed as mean and standard deviation of percentage. Asterisks represent relevant statistical difference between groups. Data is representative of three experimental sets performed individually with n = at least three mice per group in each experimental run.

To investigate the impact of macrophage polarization on the pathogenesis of ehrlichiosis, we analyzed the phenotype and frequency of monocytes and macrophages in the peritoneum (initial site of infection), spleen (secondary lymphoid organ), and liver (major site of infection and pathology) of *E. muris*- and IOE-infected mice on day 7 p.i. using flow cytometry. We selected this time point because it coincides with the difference in adaptive immune responses and outcome of infection (i.e. bacterial burden and pathology) between the *E. muris*- and IOE-infected mice as shown in previous studies^3–5^. The following cell surface markers; CD11b, F4/80 and CD68 were used to analyze: (i) non-resident or infiltrating monocyte-derived macrophages being considered as CD11b^hi^F4/80^lo^CD68^−^ cells and; (ii) Kupffer cells, which were considered as CD11b^lo^F4/80^hi^CD68^+^ cells. Gating strategy and differential expression of CD68 in the surface of these two considered populations are shown in Fig. 1A,C.

Flow cytometry analysis revealed a relevant increase in the percentage of infiltrating non-resident macrophages in the liver of both *E. muris* and IOE-infected mice, when compared to naive animals (Fig. 1A,D). However, compared to *E. muris*-infected mice, IOE-infected mice presented nearly 2-fold increase in the percentage of non-resident macrophages. While the two types of infections accounted for a relevant influx of macrophages toward the hepatic tissue, these populations were different in terms of polarization phenotypes. Under IOE infection, monocyte-derived macrophages were characterized by higher expression of intracellular iNOS (nearly 9%), which is consistent with macrophage polarization into the pro-inflammatory M1 phenotype. Lethal IOE infection was also associated with increased numbers of hepatic non-resident macrophages producing granzyme B (nearly 9%) compared to uninfected mice. In contrast, non-lethally *E. muris*-infected mice showed a small number of infiltrating macrophages expressing iNOS or Granzyme B, which was similar to that detected in the naive controls (Fig. 1B,D). Interestingly, we found that lethal IOE infection resulted in decreased percentage of Kupffer cells marked as CD11b^lo^F4/80^hi^CD68^+^ cells when compared to naive and *E. muris* infected mice. However, there was no significant difference in the levels of iNOS or granzyme B expression in Kupffer cells from all studied groups (Fig. 1D). In addition, we did not detect significant changes in the macrophage polarization in the spleen of IOE or *E. muris* infected mice, (see Supplementary Fig. S2).

**Figure 2:**
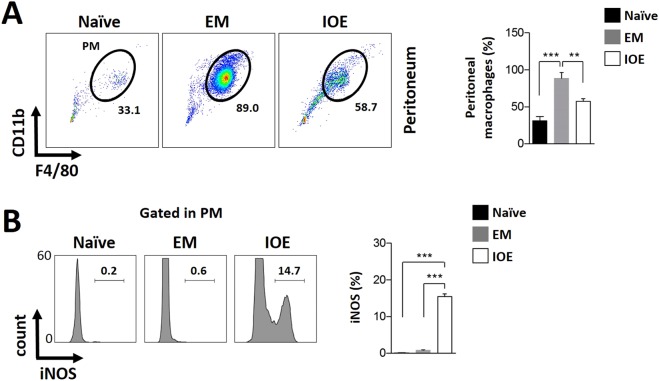
Flow cytometry analysis of the peritoneal exudate cells from *Ehrlichia*-infected mice. **(A)** Flow cytometry gate strategy for the analysis of the peritoneal exudate cells were carried out as in Fig. 1. CD3-negative cells were plotted as CD11b × F4/80 for the analysis of peritoneal macrophages defined as F4/80^+^CD11b^+^ cells. (**B**) F4/80^+^CD11b^+^ cells were also addressed for the intracellular expression of iNOS as represented by flow histograms. Values represent percentages in the assigned regions. Quantification of the analyzed cell sub-populations in the different studied groups, naive, *E. muris* (EM) and *Ixodes Ovatus Ehrlichia* (IOE) are shown in each case. Values are expressed as mean and standard deviation of percentage. Asterisks represent relevant statistical difference between groups. Data is representative of three experimental sets performed individually with n = at least three mice per group in each experimental run.

Notably, infection of C57Bl/6 mice with either *E. muris* or IOE increased the frequencies of CD11b^+^F4/80^+^ cells (considered as the macrophage population) in the peritoneal cavity, when compared to uninfected controls (Fig. 2A). However, *E. muris*-infected animals showed higher percentage levels (near 2.8-fold increase) of CD11b^+^F4/80^+^ cells in the peritoneal exudate when compared to IOE infection. Peritoneal macrophages in *E. muris*-infected mice did not polarize into M1 phenotype as marked by basal levels of iNOS expression compared to naive mice. In contrast, IOE infection resulted in a substantial increase in the percentage of peritoneal macrophages expressing iNOS compared to naive and *E. muris*-infected mice (15% vs 0.2% and 0.5%, respectively) (Fig. 2B). Together, these data suggest that IOE, but not *E. muris*, infection is associated with polarization of macrophages towards M1 phenotype in peripheral organs (peritoneum and liver) with significant expansion of infiltrating M1 macrophages in the liver, which is consistent with the development of a highly inflammatory hepatic microenvironment during fatal ehrlichial infection. On the other hand, infection with mildly virulent *E. muris* appears to induce macrophage polarization into M2 phenotypes. This assumption is also supported by the differential expansion of arginase1-expressing macrophages in the liver of *E. muris*-infected mice but not in IOE-infected animals (see Supplementary Fig. S3). This fact may account for the anti-inflammatory or regenerative tissues observed during mild *Ehrlichia* infection.

### Infection by IOE, but not by *E. muris*, induces Th17 response

**Figure 3:**
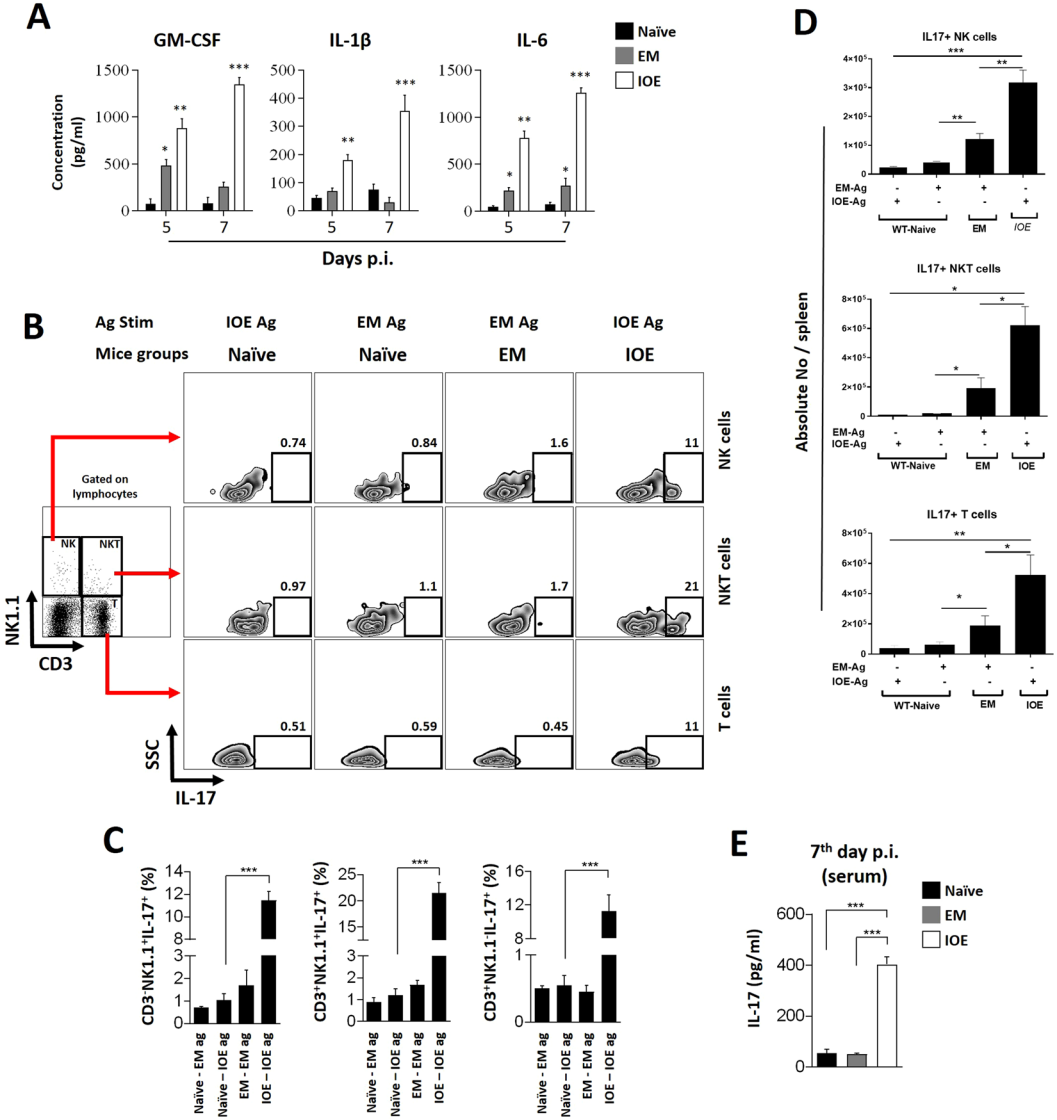
Analysis of Th17 response in *Ehrlichia*-infected C57BL/6 mice. Wild type C57BL/6 mice were intraperitoneally infected either with *E. muris* (EM) or *Ixodes ovatus Ehrlichia* (IOE) species. Serum and splenocytes were isolated at the 5^th^ or 7^th^ day post infection (p.i.) and analyzed by ELISA or flow cytometry techniques. (**A**) Quantification of pro-inflammatory cytokines related to Th17 differentiation (GM-CSF, IL-1 *β* and IL-6) in serum as measured by ELISA assay. Asterisks represent relevant differences between samples and uninfected controls. (**B**) Flow cytometry analysis of *ex-vivo* experimental sets, in which splenocytes isolated from infected or uninfected mice were stimulated with ehrlichial antigens. Cells from IOE-infected animals were treated with IOE sonicate (IOE - IOE ag) and cells from EM-infected animals were stimulated with EM sonicate (EM - EM ag). Cells from naive controls were either incubated with IOE (naive - IOE ag) or EM (naive - EM ag) antigens. After gating on splenocytes, NK1.1 × CD3 flow cytometry plots were built and three regions of analysis were considered for the assessment of intracellular IL-17 expression (evidenced in SSC × IL-17 zebra plots): NK1.1^+^CD3^−^ (conventional NK cells), NK1.1^+^CD3^+^ (NKT cells); and CD3^+^NK1,1^−^ (considered as T cells). Values represent percentage that correspond to the population in the assigned regions. Quantitative analysis of IL-17^+^ cells in the considered cell subpopulations as percentages (**C**) and absolute numbers (**D**). (**E**) Serum levels of IL-17 measured by ELISA at the 7^th^ day p.i. All quantitative analyses are expressed as mean and standard deviation. Asterisks represent relevant statistical difference between groups. Data is representative of at least three independent experiments.

Our previous studies have shown that lethal IOE infection is associated with week Th1 responses, while *E. muris* infection induces strong type 1 cell-mediated immune responses. Studies have shown that M1 macrophages’ effector functions promote the induction of inflammatory Th17 response^23^. Considering this background, we investigated whether predominance of M1 macrophages in IOE-infected mice bias the adaptive immune response towards Th17 phenotype. We observed that IOE-infected mice have higher serum levels of pro-inflammatory cytokines; GM-CSF, IL-1 *β*, and IL-6, which are known to promote M1-macrophage-Th17 axis, when compared to *E. muris*-infected or uninfected mice. Compared to uninfected mice, the serum levels of GM-CSF in IOE-infected mice were increased by approximately 12 fold on day 5 p.i., and 20 fold on day 7 p.i. Infection of mice with *E. muris* induced higher levels of GM-CSF in serum (near 5-fold increase compared to uninfected controls) on day 5 p.i., however, GM-CSF levels decreased on day 7 p.i. (Fig. 3A). Infection by IOE also led to significant increased serum levels of IL-1 *β* and IL-6 following a similar pattern as observed with GM-CSF when compared to uninfected and *E. muris*-infected mice on days 5 and 7 p.i. (**p < 0.01 and ***p < 0.001, respectively) (Fig. 3A). While *E. muris* infection significantly increased serum level of IL-6 (*p < 0.05) compared to controls on days 5 and 7 p.i. (Fig. 3A right), no significant differences were found in the levels of IL-1 *β* at both time points (Fig. 3A middle).

We previously showed that fatal IOE infection is linked to defective CD4 Th1 responses and correlates with expansion of pathogenic CD8 T cells, contributing to impaired immunity and immunopathology, respectively. Since we detected high production of pro-inflammatory cytokines and growth factors that mediate induction of Th17 response, we examined whether expansion of M1 cells in IOE-, but not E. muris-infected mice, would promote differentiation of innate immune cells such as NK, NKT cells or T cells into IL-17-producing cells in the spleen. To this end, splenocytes were isolated from infected or uninfected mice and cells were stimulated *in vitro* for 16 h with the relevant *Ehrlichia* antigen (*E. muris* or IOE) prior to assessing the intracellular expression of IL-17. Lymphocyte subsets were defined based on the expression of CD3 and/or NK1.1. Flow cytometry analyses showed that naive NK (CD3^−^NK1.1^+^), NKT (CD3^+^NK1.1^+^) and T (CD3^+^NK1.1^−^) cells stimulated *in vitro* with either *E. muris* or IOE antigens produce basal levels of IL-17 expression. On the other hand, splenocytes from IOE-infected mice showed significantly higher frequencies of IL-17-producing NK, NKT, and T cells (14-, 19-, and 18-fold increase, respectively) when compared to naive controls. In contrast, the percentage of IL-17-producing spleen NK, NKT and T cells from *E. muris*-infected mice were non-statistically significant from that detected in naive controls (Fig. 3B,C). Consistent with the percentage values, the absolute number of IL-17 producing NK, NKT, and T cells in IOE-infected mice were significantly higher than that detected in uninfected and *E. muris*-infected mice (Fig. 3D). The high magnitude of IL-17 responses was associated with the presence of statistically higher levels of IL-17 (8-fold) in the sera of IOE-infected mice on day 7 p.i., when compared to levels of IL-17 in serum from uninfected mice (Fig. 3E). On the other hand, the serum levels of IL-17 in *E. muris*-infected mice did not statistically differ from the naive group at the same time point. Together, these data suggest that IOE infection, but not *E. muris*, triggers induction and expansion of NK, NKT and T cells producing IL-17.

### IOE infection promotes polarization of bone marrow-derived macrophages into M1-type

**Figure 4:**
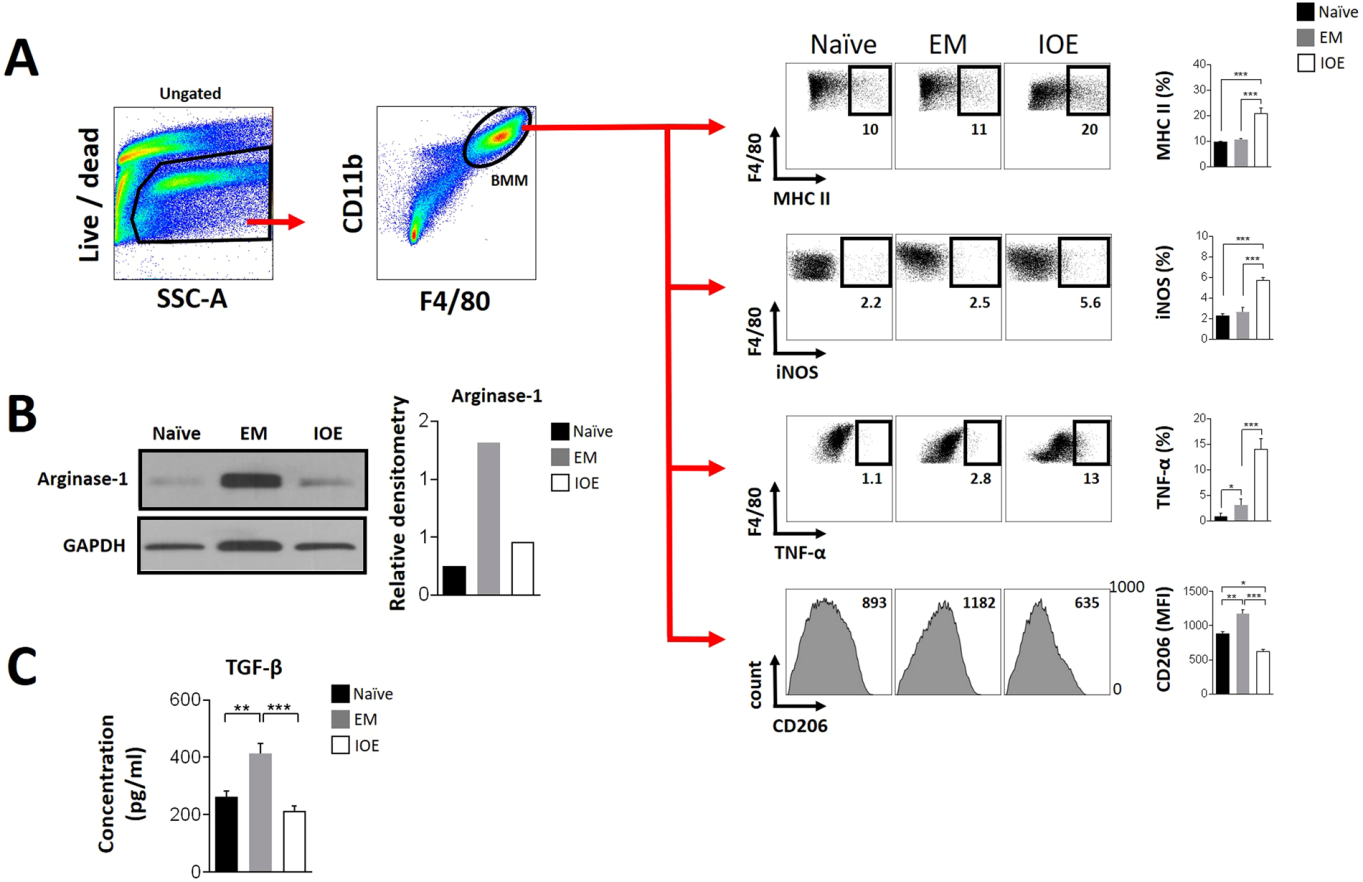
*In-vitro* analysis of bone marrow-derived macrophages (BMM) polarization upon infection with IOE or *E. muris*. BMM differentiated from bone marrow cells obtained from naive C57BL/6 mice were kept under non-polarizing conditions and infected with IOE or *E. muris* (EM). After 24 h, cells were harvested and analyzed by flow cytometry using specific markers of M1/M2 polarization. (**A**) Flow cytometry analysis of BMM after infection with *Ehrlichia*. Only CD11b^+^F4/80^+^ live cells (BMM region) were considered in this analysis as shown by the gating strategy represented by flow dot plots. Quantity of MHC class II^hi^, iNOS^+^, TNF-*α*^+^ cells (as percentage) and CD206 (as mean fluorescence intensity - MFI) were assessed within the BMM region. Each marker is accompanied by quantitative analysis considering each studied group (naive, EM or IOE). (**B**) Representative western blot analysis of protein extracts showing expression of arginase-1, and GAPDH as a load control. Quantitative analyses expressed as relative densitometry was calculated based on GAPDH levels. Full-length blots are presented in Supplementary Fig. S5, from which data were cropped and assembled. (**C**) Measurement of TGF-*β* (pg/ml) on the conditioned media of the BMM cultures infected or not with *Ehrlichia* (time point of 24 h). All quantitative analyses are expressed as mean and standard deviation. Asterisks represent relevant statistical difference between groups. Data is representative of three experimental sets performed individually.

Studies have shown that macrophage polarization occurs under M1 or M2 priming conditions mediated by LPS and IFN-*γ* for M1 polarization, and IL-4 and IL-13 for M2 polarization^32^. However, few studies investigated macrophages polarization by pathogens, which represents the initial interaction between the bacteria and the host before recruitment of effector immune cells. Thus, we examined whether IOE or *E. muris* infection could polarize primary macrophages *in vitro* without prior priming to M1 or M2 phenotypes. Bone marrow-derived macrophages (BMM) were cultured under non-polarizing conditions and infected with either IOE or *E. muris* at MOI of 5. At 24 h, cells and conditioned media were collected for flow cytometry, Western blot, or ELISA analyses. Characterization of CD11b^+^F4/80^+^ BMM as M1 by flow cytometry was determined by high expression of MCH–II (MHC-II high), iNOS and TNF-*α*, while characterization of M2 cells was defined based on high expression of CD206. Our data indicated that IOE infection increased the frequencies of MHC class II^hi^ cells (2-fold increase), while downregulating CD206 (near 2-fold decrease) on BMM, when compared to uninfected controls. Further, IOE infection induced statistically higher percentage levels of iNOS^+^(near 2-fold) and TNF-*α*^+^ cells (near 2-fold), when compared to uninfected controls. A co-expression analysis of MHC class II^+^TNF-*α*^+^ in BMM cells confirmed the observed phenotypical alteration upon infection with IOE (see Supplementary Fig. S4). In contrast, *E. muris* infection led to upregulation of CD206 (near 1.3-fold increase), while no significant changes in the expression of MHC class II or iNOS on BMM were detected when compared to uninfected BMM (Fig. 4A). Although, *E. muris* infection induced a 2.5-fold increase in the percentage of TNF-*α*^+^ BMM compared to controls, the effect of IOE was statistically higher (near 4.6-fold) in comparison to *E. muris* (Fig. 4A). Further, infection of BMM with *E. muris*, but not IOE, increased expression of arginase-1, a marker of M2 cells, as evidenced by immunoblotting analysis (Fig. 4B). Immunoblots were performed using total cell extracts, however, as the purity of CD11b^+^F4/80^+^ after the *in-vitro* differentiation was near 95%, results from total cell extracts most likely represent what is being expressed by CD11b^+^F4/80^+^ cells. Analysis of the conditioned media obtained after 24 h of infection showed 2-fold increased levels of TGF-*β* in *E. muris*-conditioned media compared to media from uninfected or IOE-infected BMM (Fig. 4C). These data suggest that under non-polarizing conditions, BMM can assume pro-inflammatory M1 phenotype under IOE infection, while infection with *E. muris* polarizes BMM towards anti-inflammatory M2 phenotypes.

### mTORC1 signaling regulates macrophage polarization in BMM infected with IOE

**Figure 5:**
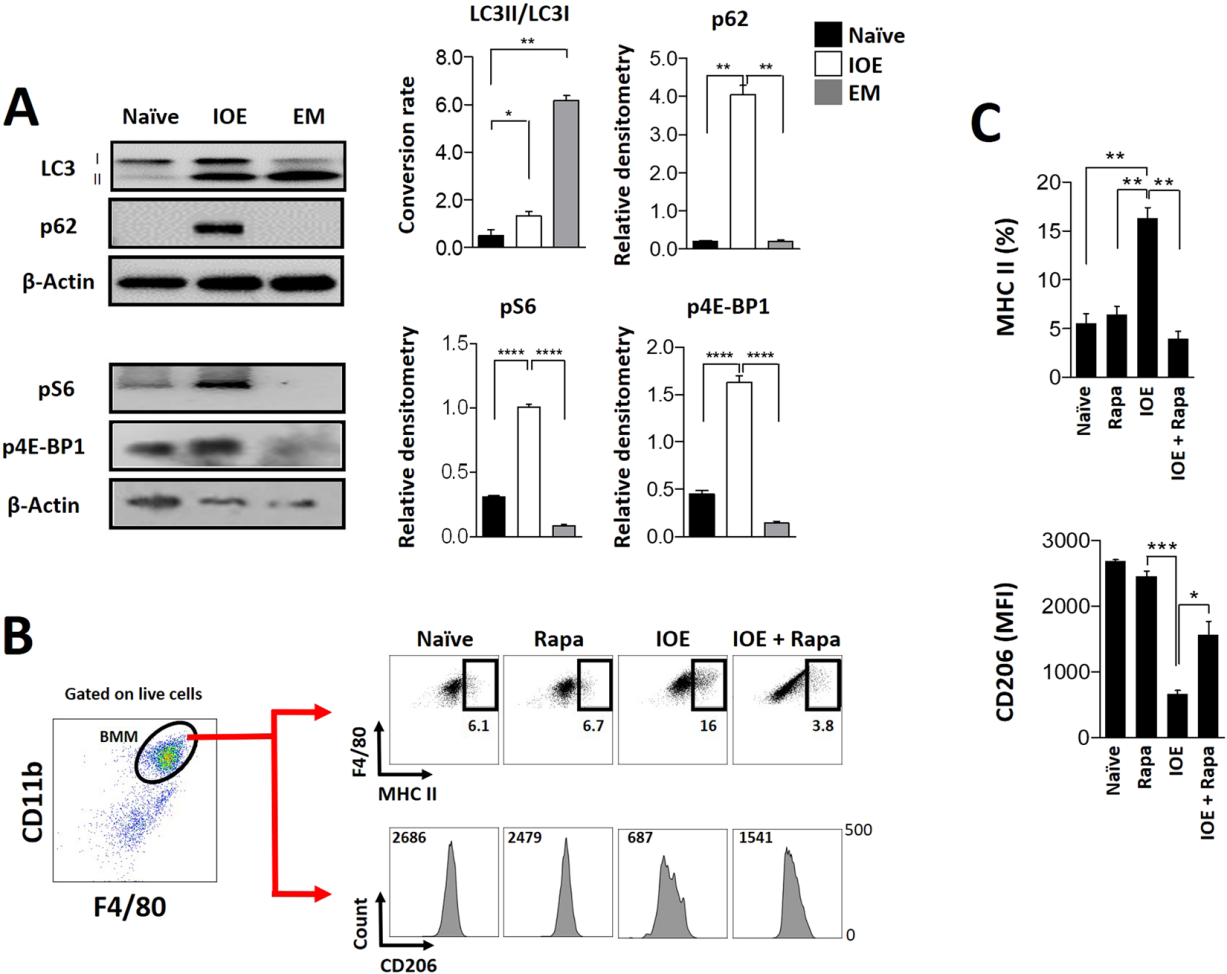
Assessment of autophagic activity and mTORC1 signaling in macrophage polarization during infection with *Ehrlichia*. Assessment of autophagic activity in bone marrow-derived macrophages (BMM) infected with *E. muris* or IOE was carried out by western blot analysis. BMM were incubated or not with bacteria for 24 h prior to obtaining protein extracts from the different studied groups (*E. muris*-, IOE-infected and uninfected BMM). Flow cytometry was used to assess the impact of mTORC1 signaling in macrophage polarization via stimulation of BMM with mTORC1 inhibitor, rapamycin (Rapa) 10 *μ*M, by the time of infection. (**A**) Measurement of LC3I and LC3II in the protein extracts accompanied by quantitative analysis of LC3II/LC3I ratio of conversion. Ratio of conversion was assessed after normalization of densitometry values based on the levels of *β*-actin. Levels of p62 and downstream targets of mTORC1 signaling, pS6 and p4E-BP1, in the considered protein extracts are also shown. Quantitative analyses expressed as relative densitometry are shown for each marker. Full-length blots are presented in Supplementary Fig. S6, from which data were cropped and assembled. (**B**) Flow cytometry dot plot showing region of considered BMM (CD11b^+^ F4/80^+^ cells gated on live cells) in which the percentage of MHC class II^high^-expressing cells and the mean fluorescence intensity (MFI) of surface CD206 were measured. Values near the regions represent percentage and numbers above histograms indicate MFI values. (**C**) Quantitative analysis for flow cytometric determinations. All quantitative analyses are exhibited as mean and standard deviation. Asterisks represent relevant statistical difference between groups. Data is representative of at least two independent experiments.

We recently showed that fatal IOE infection triggers activation of mTORC1, a negative regulator of autophagy. Activation of mTORC1 inhibit autophagy induction and flux and causes inflammasome activation, which in turn promotes dysregulated inflammation and liver injury. We hypothesized that IOE-induced polarization of macrophages towards M1 phenotype could be linked to mTORC1 activation. To test this hypothesis, we first assessed the induction of autophagy in IOE-infected BMM (M1 cells) or *E. muris*-infected BMM (M2 cells). To examine autophagy induction, we analyzed the conversion of LC3I to LC3II as a marker of autophagosome formation using western blot analysis. Our data show that there is an increase in the LC3II:I ratio of conversion in IOE-infected BMM compared to uninfected cells (p ≤ 0.05). However, the LC3II:I ratio of conversion in IOE-infected BMM was significantly lower (p ≤ 0.01) than that detected in *E. muris*-infected BMM, suggesting significantly lower levels of autophagy induction in IOE-infected BMM in comparison to *E. muris*-infected BMM (Fig. 5A). To further examine autophagic flux, we analyzed the level of p62/SQSTM1, a selective autophagy adaptor/receptor that binds to ubiquitinylated proteins and damaged organelles to target them to autophagosome-lysosomal compartments for degradation. The total cellular p62 expression levels inversely correlate with the autophagic flux and activity. Our results showed significant increase in p62 levels in IOE-infected cells compared to uninfected or *E. muris*-infected cells. Autophagic flux was also investigated by confocal microscopy examining the co-localization of LC3 puncta with acidic organelles (see Supplementary Fig. S7). To this end, cells were stained with an antibody against LC3 and also with LysoTracker Red, an acidotropic fluorescent dye that accumulates in acidic lysosomes. Compared to IOE-infected BMM, *E. muris*-infected BMM exhibited an evident qualitative increase in the number of LC3 puncta per cell that colocalized with the LysoTracker Red, suggesting formation of autolysosomes (see Supplementary Fig. S7). Decreased autolysosome formation in IOE-infected BMM was not due to a block in lysosomal acidification by IOE because the number of LysoTracker Red staining in IOE-infected and EM-infected cells was similar. Thus both confocal microscopy and immunoblotting analyses indicated that IOE induces blockage of the autophagosome-lysosomal fusion/autophagic flux.

We next investigated the mTORC1 activity which is determined by the expression of phospho-S6 (pS6) and phospho-4E-BP1 (p4E-BP1); downstream substrates of active mTORC1 pathway. IOE-infected BMM showed significantly higher levels of pS6 and p4E-BP1 when compared to *E. muris*-infected or uninfected BMM. Expression of either pS6 or p4E-BP1 during *E. muris* infection was completely abrogated, suggesting suppression of mTORC1 activity in macrophages following *E. muris* infection (Fig. 5A). To further confirm the participation of mTORC1 in the promotion of M1 polarization following IOE infection, we infected BMM with IOE in the presence or absence of rapamycin (Rapa), which is a well-known inhibitor of mTORC1 activity. In this case, the frequencies of MHC class II^hi^ cells and surface expression of CD206 were taken into account to define M1 induction in these cells. We observed that control BMM (uninfected BMM or cells incubated with Rapa only) show low percentages of MHC class II^hi^ cells (near 6–7%). As expected, infection with IOE led to increased percentages of MHC class II^hi^ BMM (near 2.5-fold compared to controls). Culture of IOE-infected BMM with rapamycin (10 *μ*M) resulted in significantly lower percentages of MHC class II^hi^ cells when compared to IOE infection (Fig. 5B,C). The same effect was observed when analyzing the expression of CD206. IOE-infected BMM treated with rapamycin expressed higher levels of CD206 when compared to untreated IOE-infected BMM (near 2-fold increase) (Fig. 5B,C). Together, these data suggest that IOE-induced polarization of macrophages towards M1 phenotype is due to mTORC1 activation, while M2 polarization following *E. muris* infection is linked to suppression of mTORC1 activation.

## Discussion

In this study, we investigated the contribution of dysregulated effector functions involving macrophage polarization to the pathogenesis of Ehrlichiosis. Using murine models of lethal and nonlethal ehrlichiosis, we showed that lethal *Ehrlichia*-induced sepsis is associated with accumulation of infiltrating pro-inflammatory M1 macrophages/monocytes in the liver tissue. In contrast, mild ehrlichiosis is associated with polarization of macrophages into M2 phenotypes, which are important in the resolution phases of inflammation. Consistent with *in vivo* findings, *in-vitro* investigation revealed that infection by highly virulent IOE or mildly virulent *E. muris* species induce polarization of BMM into distinct types. Mechanistically, we found that M1 polarization during IOE infection is linked to activation of mTORC1 signaling and that blockade of mTORC1 by rapamycin attenuated M1 polarization following IOE infection.

The correlation between expansion of M1 cells in IOE-infected mice with development of immunopathology and sepsis, as well as correlation of M2 with induction of protective immunity following *E. muris* infection, suggest that macrophage phenotypes may influence the outcome of infection. M1 macrophages are known to promote inflammation in attempt to clear intracellular pathogens as suggested by other infection models^33–35^. Once infection is cleared, the immune responses mount a regulatory mechanism that dampen inflammation and prevent unwanted side effects such as autoimmunity or immunopathology. However, under conditions where host fail to eliminate pathogens due to impaired protective immunity, the persistence of the inflammatory stimuli can lead to tissue damage in targeted peripheral organs^36^. This is essentially what we observed in *Ehrlichia*-induced sepsis, in which accumulation of monocyte-derived macrophages in the liver of infected mice coincided with organ damage and dysfunction. While upregulation of iNOS and Granzyme B by these cells characterized their microbicidal activity, it could also be a cause of organ dysfunction since increased expression of these markers have also been associated to damage in the hepatic tissue^37–40^. Lower amounts of Kupffer cells found in the IOE-infected mice, when compared to controls, also highlighted that the infection would lead to limited protective mechanisms by resident cells. Conversely, infection with *E. muris* led to mild accumulation of macrophages in the peritoneal cavity, which is the initial site of infection. Our finding that these peritoneal macrophages are iNOS-negative is consistent with limited inflammation and minimal tissue injury observed in the peripheral organs of *E. muris*-infected mice. Still considering the *in-vivo* scenario, we previously showed that mice infected with IOE at a dose of 1–5 × 10^3^ bacteria/mouse have higher bacterial burden in the peritoneum at 4 hours post infection than mice infected with *E. muris* at 5–10 × 10^6^ bacteria/mouse^31^. The high bacterial burden in IOE infected mice at early time points (4 h and 24 h) was not due to macrophage cell death since we detected a significant expansion of macrophages in peritoneum of these mice compared to *E. muris*-infected mice. From a big picture, it is important to note that IOE survives and/or replicates in mice significantly better than *E. muris* (see Supplementary Fig. S1). These differences in bacterial replication/physiology could also represent an important factor to influence the host immune response and account for distinct aspects regarding cell infiltration and polarization.

Macrophages are critical not only in innate immunity, but also in the activation of acquired immune responses. Krausgruber and coworkers found that M1 macrophages promote Th1-Th17 response via interferon regulatory 5 (IRF5) signaling^23^. Moreover, as recently suggested by Nakai and coworkers, once the Th17 response is established, IL-17 could also play a role in enhancing macrophage differentiation into M1 type^41^, which highlights the existence of a possible positive feedback loop involving M1-Th17 axis. While this M1-Th17 responses can mediate pathogen clearance, they can exacerbate inflammation and cause tissue damage^36^. Our data showed that IOE, but not *E. muris*, infection induced high levels of IL-17 in the serum of mice on day 7 p.i. Additionally, IOE-infected animals showed increased serum levels of pro-inflammatory cytokines known to promote M1-Th17 differentiation such as GM-CSF, IL-1 *β*, and IL-6. GM-CSF is known to polarize macrophages into M1-type^42^, while IL-1 *β* and IL-6 support induction and expansion of antigen-specific Th1 and Th17 cell responses. In this study, we found that not only T cells, but also conventional NK, and NKT cells contribute to the production of IL-17 during IOE-infection. Since such responses were not seen in mice infected with *E. muris*, it is possible that M1-Th17 axis would play a role in the pathogenesis of fatal ehrlichiosis by promoting inflammation and host cell death. Yet, future studies will investigate in detail the contribution of Th17 to host defense against *Ehrlichia* and disease pathogenesis.

Considering the polarization of macrophages under an *in-vitro* approach, these cells are routinely shifted into M1 or M2 types by providing a set of specific stimuli^43^. For example, IFN-*γ* and lipopolysaccharide (LPS) stimulation promote polarization into the M1-macrophage type, while IL-4, IL-10, IL-13 promote M2 polarization. However, under non-polarizing conditions, bacterial infections alone stimulate macrophages to respond with common transcriptional activation programs, which generally involve upregulation of genes for M1 polarization^44–46^. M1-macrophage phenotype is often characterized by the upregulation of MHC class II, downregulation of manose receptor (CD206) cell surface, as well as intracellular over-expression of iNOS and TNF-*α*, which are involved in microbicidal activity and induction of inflammation, respectively^22,47,48^. On the other hand, M2-macrophage phenotypes (which comprise M2a, M2b and M2c) are usually defined by the upregulation of CD206, arginase-1, IL-10 and TGF-*β*, which correlate with tissue repair and suppressive or non-damaging roles^46,49–51^. Our *in-vitro* approach indicates that IOE or *E. muris* were able to shift BMM into distinct macrophage types, which was consistent with our *in-vivo* data. IOE-induced polarization of macrophages into M1 phenotype is rather intriguing since *Ehrlichia* species do not express LPS, which is a major pathogen-associated molecular pattern (PAMP) that primes macrophages into M1 phenotype. Further, cells infected with either IOE or *E. muris* do not produce significant levels of IFN-*γ*, IL-4, and IL-10 that could possibly promote M1/M2 polarization via autocrine/paracrine signals. Nevertheless, how IOE or *E. muris* trigger M1 and M2 polarization, respectively, and what are the ehrlichial PAMPs involved in these processes remain elusive.

Our data demonstrate an interesting connection between the macrophages’ autophagic activity and polarization into distinct types. Studies in cancer models have shown that activation of autophagy regulates polarization of macrophages into the M2-type^15,28^, although the link with M1 or M2 was context-dependent. For example, deficiency of Atg5, a major protein required for initiation of autophagy^52^, induces M2 polarization. Other studies also supported that inhibition of autophagy attenuates M2 macrophage polarization^53,54^. In our study, we found that *E. muris*-induced M2 macrophages exhibit enhanced autophagy, in comparison to IOE-induced M1 macrophages. The differences in autophagy process was linked to mTORC1 activation in IOE-, but not in *E. muris*-infected cells. Inhibition of mTORC1 signaling with rapamycin indicated that mTORC1 modulates M1 macrophage polarization upon IOE infection. Our model system and data revealed a complex interplay between host cell metabolism mediated by mTORC1 and intracellular bacterial survival and replication. Previous studies by us and other investigators have indicated that IOE and *Ehrlichia chaffeensis* exploit autophagy proteins to obtain nutrients for their survival and replication. However, our data suggest that activation of mTORC1 by IOE, but not autophagy induction, is required for IOE replication. Since mTORC1 is a key regulator of lysosome structure and function, it is possible that inhibition of mTORC1 by *E. muris* organisms promotes not only enhanced autophagy, but also increased lysosome biosynthesis and autophagic flux. The latter could account for restricted intracellular growth of *E. muris*. On the other hand, activation of mTORC1 upon IOE infection may not only promote host cell survival that is required for survival of the obligate intracellular virulent *Ehrlichia*, but also inhibition of autophagic flux. Enhanced host cell survival and blockage of autophagic flux by activating mTORC1 in IOE-infected macrophages could account for bacterial growth and survival. Our IOE model is therefore different than other obligate intracellular pathogen such as *Coxiella burnetii* where hyper activation of mTORC1 impaired bacterial replication via non canonical pathway that is independent of enhanced autophagy^55^.

The differential effect of IOE and *E. muris* on macrophage polarization suggests that the two infections are involved with distinct transcriptional activation programs, as well as with distinct mechanisms of host-pathogen recognition. Although the exact mechanism that account for these differential responses are not completely understood, it is possible that IOE and *E. muris* may be differentially sensed by macrophages. Our previous studies have shown that both Toll-like receptor 2 (TLR2) and nucleotide-binding oligomerization domain containing 2 (NOD2) receptors are upregulated in the liver during IOE, but not in *E. muris* infection^30^. Using knockout mice, we demonstrated that TLR2 signaling mediates protection, while NOD2 signaling seems to be strictly involved with the development of immunopathology in ehrlichiosis^30^. Other studies have shown that stimulation of TLR2 drives M2 polarization resulting in activated autophagic machinery^56,57^. Stimulation of NOD2 in human macrophages increases expression of pro-inflammatory cytokines driving M1 polarization^58^. In corroboration to these findings, it was recently found that macrophages polarized with IFN-*γ* to assume M1 phenotype, show induction of NOD2 only, among several other studied NOD-like receptors (NLRs)^59^. However, the correlation between activation of NOD2 and polarization towards M1-type macrophages seems to be controversial, since other studies showed that deletion of NOD2 did not produce alterations in macrophage polarization^60^. Considering that infection by IOE and *E. muris* produce distinct *in-vivo* phenotypes (fatal vs. mild disease, respectively), recognition by *Ehrlichia* involving TLR2 or NOD2 signaling in macrophages could be hypothetically correlated with the particular polarization into M1 or M2 types. Nevertheless, the role of specific sensing of *Ehrlichia* by TLR2 and NOD2 in macrophages is yet to be elucidated in order to draw conclusions about polarization in each case.

In conclusion, the two infection models, mild and fatal ehrlichiosis considered here, worked as outstanding tools to indicate distinct roles and contributions of macrophages in both disease contexts. On the one side, M2-type macrophages with homeostatic and anti-inflammatory activities may contribute to effective control of *E. muris* infection at the early stages. It is also possible that optimal elimination of intracellular bacteria by M1-type macrophages followed by timely regulation of M2-type cells may account for a mild outcome and minimal pathology following *E. muris* infection. This conclusion is supported by our data showing marginal increments in serum levels of GM-CSF and IL-6 under infection by *E. muris*. On the other side, the overwhelming and dysregulated M1-type effector functions by monocyte-derived infiltrating macrophages into peripheral tissues would contribute to immunopathology following IOE infection. These M1-type cells would further lead to a hyper-inflammatory state by promoting induction and/or expansion of antigen-specific Th17 as well as secretion of IL-17 by NK and NKT cells. This work unravel for the first time the importance of considering macrophage heterogeneity in the outcome of *Ehrlichiosis*, drawing attention to novel avenues for drug development and for the discovery of biomarkers in HME.

## Methods

### Materials and methods

#### *In-vivo* experiments

Ethics: This study was carried out in strict accordance with the recommendations in the Guide for the Care and Use of Laboratory Animals of the National Institutes of Health. The protocol for this work ACC 18–139 was approved by the Committee on the Ethics of Animal Experiments of the University of Pittsburgh in accordance with the institutional guidelines for animal welfare.

Mice and infection: Female specific pathogen free (SPF) C57Bl/6 WT mice ranging from 8 to 12 weeks old were obtained from Jackson Laboratories (Bar Harbor, ME). All animals were housed under specific pathogen-free conditions at the Animal Research Facility in the University of Pittsburgh.

Experiments in mice were carried out as in previous reports^30,31^. Briefly, the highly virulent strain of *Ehrlichia*, *Ixodes Ovatus Ehrlichia* (IOE) and the mildly virulent *Ehrlichia muris* were kindly provided by Dr. Yasuko Rikihisa (Ohio State University, Columbus, OH). IOE and *E. muris* stocks were propagated by passage through WT C57BL/6 mice via intraperitoneal (i.p.) infection of 10^4^ organisms. Splenic single-cell suspensions extracted from spleens of mice day 7 post infection (p.i.) were stored in sucrose and potassium phosphate (SPK) buffer (0.5 M K_2_HPO_4_, 0.5 M KH_2_PO_4_, and 0.38 M sucrose) and kept in liquid nitrogen. For the *in-vivo* experimental setting, mice were i.p.-infected with doses ranging from 10^3^ to 10^4^ of IOE organisms per mouse or a high dose of *E. muris* (10^4^ to 10^5^ organisms per mouse). On day 7 p.i., animals were sacrificed, blood samples were collected by cardiac puncture for serum isolation and organs (spleens and livers) were harvested for further investigation. For survival experiments, mice were monitored daily for signs of morbidity.

### Isolation and infection of bone-marrow-derived macrophages (BMM)

Bone marrow cells were isolated from naive WT mice and BMM were prepared as described previously^61^. Briefly, femurs were excised and flushed under aseptic conditions. Bone marrow cells were seeded in 100 mm petri dishes at 2 × 10^6^ cells/10 ml/dish in DMEM/F12-GlutaMAX (Invitrogen) supplemented with 10% FBS (Invitrogen), 20 ng/ml MCSF (PeproTech), 10 mM Hepes (Invitrogen), and 10 mM glutamine (Invitrogen). Approximately 4 ml of fresh media was added after 3 days of culture. On day 6, cells were collected and the purity of differentiated BMM was determined by flow cytometry using anti-CD11b surface marker. The number of CD11b^+^ cells isolated by this method was : 95%. BMM collected on day 6 were seeded into 12-well plates for 20 h prior to infection at a density of 10^6^ cells/500 *μ*l/well in DMEM/F12 (Invitrogen) supplemented with 5% FBS, 10 ng/ml MCSF, 10 mM Hepes, and 1% pen/strep. For the infection, cell-free IOE organisms were prepared from IOE-infected splenocytes as previously described^4^. Cell-free *E. muris* organisms were prepared from *E. muris*-infected DH82 cells (Canine macrophage cell line). IOE and *E. muris* organisms were added to the BMM cultures at multiplicity of infection (MOI) of 5. Cells were collected 24 h post-infection for flow cytometry analysis, or pellets were frozen for further analysis. Supernatants were also collected and stored at −80 °C for cytokine determination. For inhibition analysis with rapamycin, BMM (1.5 × 10^6^) where treated or not with rapamycin (10 *μ*M, InvivoGen) and infected or not with IOE. After 24 h, cells were collected for flow cytometry analysis following a protocol described below.

### Isolation of liver mononuclear cells (LMNCs)

LMNCs were isolated and purified using a modified enzymatic dispersal protocol as described previously^62^. Hepatocytes were removed by differential centrifugation (36 × g) for 1 minute at 4 °C, and the final pellet containing LMNCs was resuspended in RPMI 1640 medium containing 10% fetal calf serum and 1% HEPES. LMNCs were purified using Lympholyte M (Cedarlane Laboratories, Burlington, NC).

### Isolation of peritoneal exudate cells (PEC)

The peritoneal cavities of the uninfected and infected mice were washed with 10 ml sterile phosphate-buffered saline (PBS), and the peritoneal wash/lavage fluid was collected at the day 7 post infection. Cells were centrifuged at 275 × g for 5 min to separate the peritoneal exudate cell fractions (PEC).

### Flow cytometry

Splenocytes isolated by maceration of spleens, liver mononuclear cells (LMNCs) and peritoneal exudate cells (PEC) were obtained from infected or non-infected mice. Before staining with specific cell markers, samples were pre-incubated with live/dead staining Zombie Aqua^TM^ for 30 minutes following manufacturer’s instructions (cat. number 423101, Biolegend) In sequence, cells were washed and re-suspended in fluorescence-activated cell sorter-staining buffer at a concentration of 10^6^ cells/tube. FcRs were blocked with a mAb (clone 2.4G2) against mouse cell surface antigens CD16 and CD32 for 15 min. The following FITC-, PE-, PerCP-Cy5.5–, Alexa Fluor-, and APC-conjugated Abs were used (all antibodies were purchased from either BD Biosciences or Biolegend): anti-CD11b (101212), anti-F4/80 (123118), anti-CD3 (clone 145- 2C11), anti-NK1.1 (clone PK136), anti-CD68 (clone RA3-6B2), anti-MHC II (clone M5/114.15.2), anti CD206 (clone C068C2), anti-TNF-*α* (clone MP6-XT22), anti-iNOS (clone W16030C), anti-Granzyme B (clone NGZB) and anti-IL-17 (clone TC11-18H10). For intracellular cytokine staining, the cells were incubated with BD Golgi Plug. Approximately 50,000 events were acquired for the spleen cells, 10,000 for PECs the 100,000 for LMNCs using the BD-LSR and BD FACSCalibur (BD Immunocytometry Systems, San Jose, CA) flow cytometry, and the data were analyzed using FlowJo software (TreeStar, Ashland, OR).

For the assessment of Th17 response, splenocytes (2 × 10^6^ cells) from naive or infected mice were stimulated *in vitro* with *E. muris* or IOE antigens (prepared by sonication of cell-free bacteria) for 12 h to determine the frequency of antigen-specific T cells. *In-vitro* stimulated spleen cells were then harvested and immunophenotyped with anti-CD3, anti-NK1.1 and anti-IL-17 mabs by flow cytometry.

### Quantitative real-time PCR (RT-PCR) for bacterial burden

Total DNA was isolated from liver and spleen tissues using the DNeasy Blood and Tissue kit (QIAGEN). Bacterial burden was determined using an iCycler IQ multicolor real-time detection system (Bio-Rad Laboratories, Hercules, CA), primers and probes for the IOE *dsb* gene as previously described^5^. The primers and probes used were: IOE *dsb* forward: 5′-CAG GAT GGT AAA GTA CGT GTG A-3′; IOE *dsb* reverse: 5′-TAG CTA AYG CTG CCT GGA CA-3′; IOE probe: (6FAM)-AGG GAT TTC CCT ATA CTC GGT GAG GC-(MGB-BHQ). The eukaryotic housekeeping gene *gapdh* was amplified using the following primers/probes: *gapdh* forward: 5′-CAA CTA CAT GGT CTA CAT GTT C-3′; *gapdh* reverse: 5′-TCG CTC CTG GAA GAT G-3′; *gapdh* probe: (6FAM)-CGG CAC AGT CAA GGC CGA GAA TGG GAA GC-(MGB-BHQ). The comparative cycle threshold (CT) method was used to determine the bacterial burden as described previously. Results were normalized to the levels of *gapdh* expression. Samples with CT ≥ 38 in the PCR reaction were considered negative for *Ehrlichia* DNA.

### Cytokine determination

Levels of IL-1 *β*, IL-6, IL-17 and GM-CSF in serum of Naive, IOE or *E. muris* infected mice were analyzed by commercially available enzyme-linked immunosorbent assay (ELISA) (eBioscience, San Diego, CA; Vienna, Austria) kits according to the manufacturer’s instructions. TGF-*β* 1 levels in culture supernatants from uninfected, IOE- or *E. muris*-infected BMM were determined using Quantikine immunoassay kits (R&D Systems, Minneapolis, MN).

### Western blot analysis

Western blot analysis were carried out based on a previous protocol^61^. Briefly, liver tissues and BMM were lysed in T-PER lysis buffer or RIPA buffer (Thermo Fisher Scientific, Waltham, MA), respectively, supplemented with protease inhibitors and 1 mM phenylmethylsulphonyl fluoride (PMSF). Protein extraction was performed at 4 °C for 30 min. The protein content of the lysates was measured using a Bicinchoninic Acid Assay Kit (Pierce). Lysates (15–40 *μ*g) were resolved in 4% to 20% gradient SDS–PAGE under reducing conditions. The membranes were processed and probed with the following antibodies: anti-pS6 (1:2000), anti-p4E-BP1 (1:1000) (Cell Signaling technology), anti-LC3B (1:1000), anti-SQSTM1/p62 (1:1000, Cell Signaling technology) and anti-Arginase-1 (1:1000) (Novus Biologicals, Littleton). Blots were probed with the appropriate primary antibodies and peroxidase-conjugated bovine anti-rabbit secondary antibodies (1:10000) (Santa Cruz Biotechnology). Blots were stripped with Restore Western Blot Stripping Buffer (Pierce) and re-probed with anti-*β*-Actin (1:2500, Abcam). Mean pixel density of bands in Western blots was determined using ImageJ software version 1.48 (NIH, Bethesda, MD).

### Immunofluorescence staining and confocal microscopy

The number of LC3 puncta and colocalization of LC3 with acidified lysosomes was determined by confocal microscopy as previously described^61^. Briefly, BMM cultured on cover slips were infected with IOE at MOI of 5 or left uninfected. Cells were then washed 3X with PBS, fixed with 2% paraformaldehyde for 20 min, and permeabilized with 0.1% Triton X-100 in PBS for 30 min. After blocking with 5% BSA (Sigma-Aldrich, A2153) for 60 min, the primary antibodies; anti-LC3 (Sigma, 50 ug/mL) was added for 1 h at room temperature. Cells were washed and then incubated with fluorescent labeled anti-rabbit secondary antibody DyLight (VectaFluor, 1:500) for 1 h. Nuclei were stained with DAPI and cells were analyzed by confocal microscopy (Olympus Flouview 1000). Analysis of acidified lysosome was performed using LysoTracker Red (cat. L-12492, Thermofisher) at 37 °C for 1 h and assessed with a confocal microscope (Olympus flouview 1000).

### Statistical analysis

All presented data are representative of two or three independent experiments that yielded corroborative results. Two group analyses were performed using an unpaired two-tailed t test. For comparison of multiple experimental groups, we used one–way analysis of variance (ANOVA) with Bonferroni’s procedure. Calculations were processed using Graph Pad Prism (GraphPad Software Inc., La Jolla, CA, USA) and values are expressed as mean and standard deviations (SD). Differences between groups that were statistically significant were represented as: * for *p* values ≤ 0.05, ** for *p* values ≤ 0.01, *** for *p* values ≤ 0.001 and **** for *p* values ≤ 0.0001.

## Supplementary information

Supplementary information

## Data Availability

All data generated or analyzed during this study are included in this published article.
