# Supplementary material for: mTORC1-mediated polarization of M1 macrophages and their accumulation in the liver correlate with immunopathology in fatal ehrlichiosis
Source: Sci Rep. 2019 Oct 1;9:14050. doi: 10.1038/s41598-019-50320-y (PMC6773708; doi:10.1038/s41598-019-50320-y)
Supplement: Supplementary file 1 — Supplementary information [file 41598_2019_50320_MOESM1_ESM.pdf]

Supplementary Information

mTORC1-mediated polarization of M1 macrophages and their accumulation in the liver correlate with immunopathology in fatal ehrlichiosis

Mohamed Haloul[1,2], Edson R. A. Oliveira[1], Muhamuda Kader[3], Jakob Z. Wells[3], Tyler R. Tominello[3], Abdeljabar El Andaloussi[1], Cecelia Yates[4], and Nahed Ismail[1,*]

[1]Department of Pathology, College of Medicine, University of Illinois at Chicago, Chicago, IL, USA

[2]Children's Cancer Hospital Egypt 57357, Cairo, Egypt

[3]Department of Pathology, School of Medicine, University of Pittsburgh, Pittsburgh, PA, USA

[4]Nursing School, University of Pittsburgh, Pittsburgh, PA, USA

*Correspondent author: ismail7@uic.edu

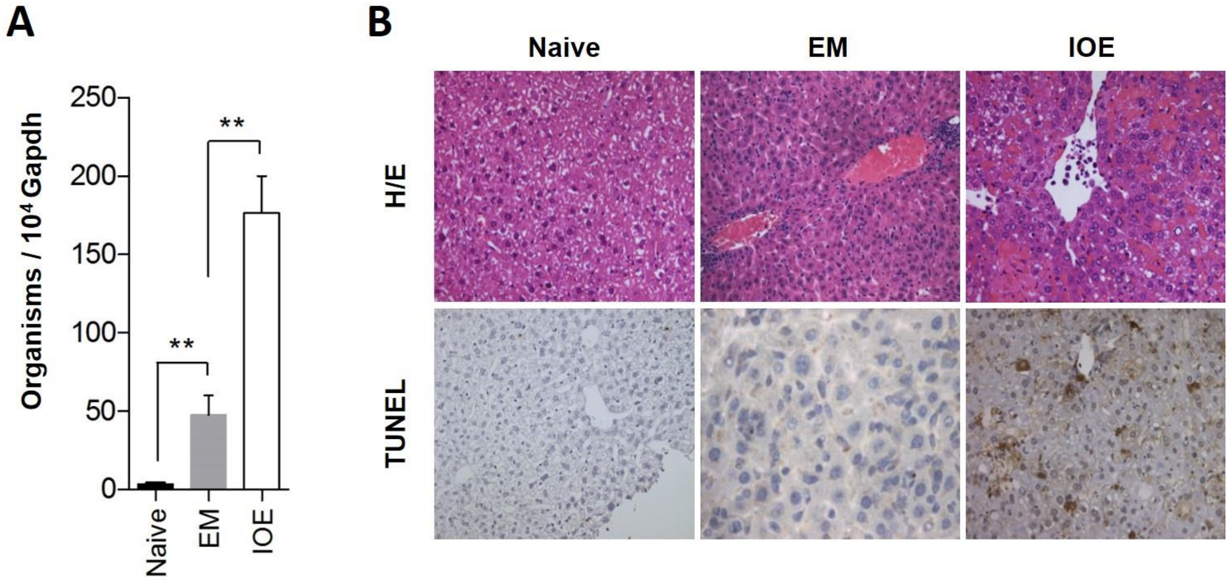

S1 Fig. Bacterial burden and liver damage caused by *Ehlichia*. **A.** Bacterial burden in the liver of EM- and IOE-infected mice found at the 7th day p.i. as measured by quantitative PCR. The gene considered for amplification and determination of bacterial burden was the *dsb* gene. Results were normalized by the expression of *Gapdh* housekeeping gene. **B.** Hematoxylin and eosin (H/E) staining showing aspects in the liver tissue of infected animals (top) and immunohistochemistry (IHC) for assessment of late-stage apoptosis by TUNEL assay (bottom).

Methods:

Histopathology and terminal deoxynucleotidyl transferase dUTP nick end labeling (TUNEL) assay

Liver sections were fixed in a 10% solution of buffered formalin, dehydrated in graded alcohols, embedded in paraffin wax, and stained with hematoxylin and eosin (H/E). TUNEL staining was performed on unstained tissue sections, showing apoptotic cell death without focal necrosis, as described previously[1].

[1]Ismail, N. et al. Overproduction of TNF-alpha by CD8+ type 1 cells and down-regulation of IFN-gamma production by CD4+ th1 cells contribute to toxic shock-like syndrome in an animal model of fatal monocytotropic ehrlichiosis. J Immunol 172, 1786–1800 (2004).

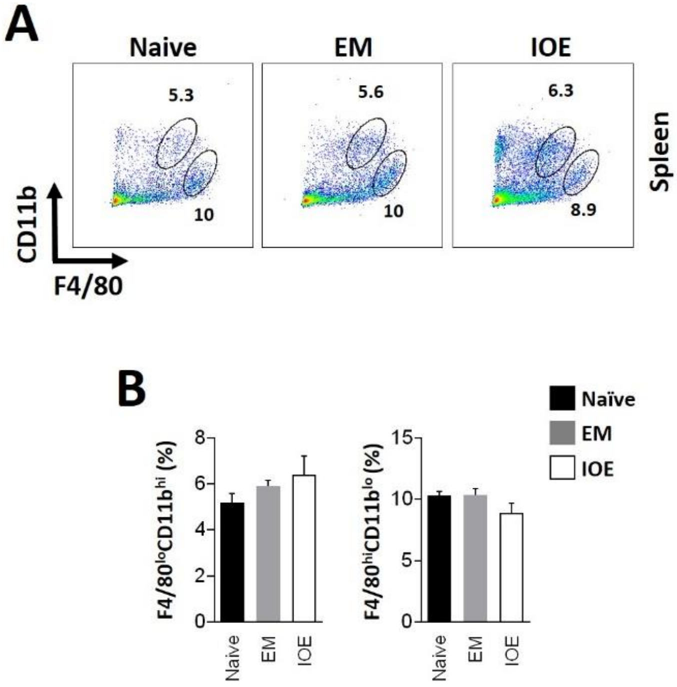

S2 Fig. Flow cytometry analysis of the spleen from *Ehrlichia*-infected mice. A. Flow cytometry gate strategy for the analysis of the splenocytes was carried out as in Fig. 1 present in the main text of the manuscript. CD3-negative cells were plotted as CD11b vs F4/80 for the analysis of infiltrating macrophages/monocytes defined as F4/80^{lo}CD11b^{hi}cells, and the resident macrophages, considered as F4/80^{hi}CD11b^{lo}cells. **B.** Quantification of the analyzed cell sub-populations in the different studied groups, naive*, E. muris* (EM) and *Ixodes Ovatus Ehrlichia* (IOE) are shown in each case. Values are expressed as mean and standard deviation of percentage. Data is representative of three experimental sets performed individually with n = at least three mice per group in each experimental run. No statistical differences were found between groups.

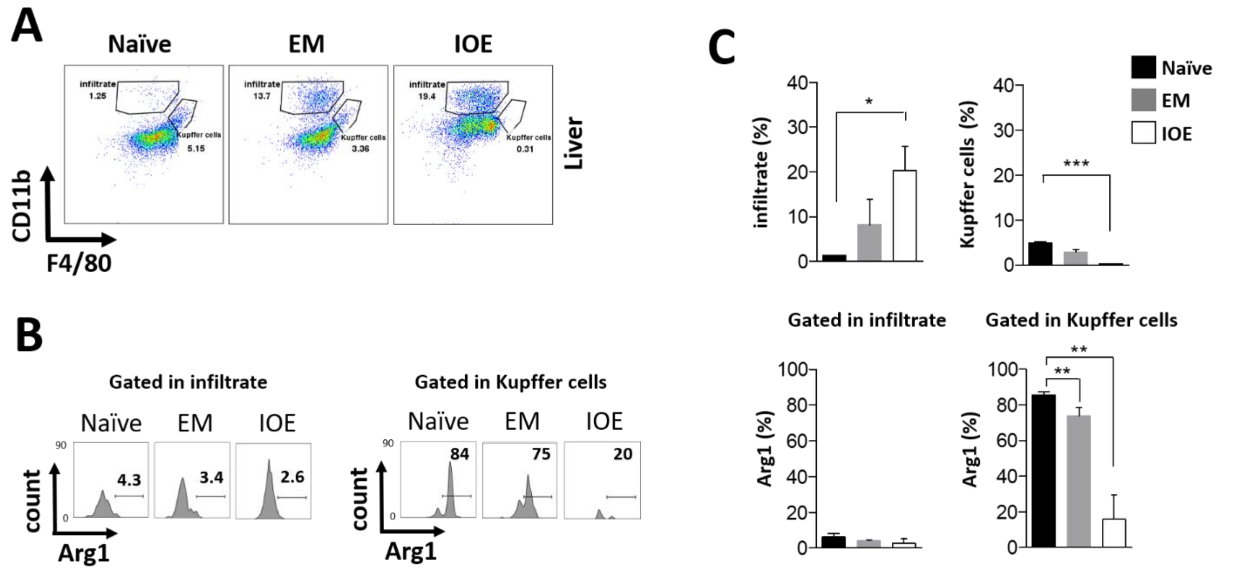

S3 Fig. Flow cytometry analysis of the liver from *Ehrlichia*-infected mice for the investigation of M2-macrophage markers. **A.** Flow cytometry gate strategy for the analysis of the liver cells was carried out as in Fig. 1 present in the main text of the manuscript. CD3-negative cells were plotted as CD11b vs F4/80 for the analysis of infiltrating macrophages/monocytes defined as F4/80^{lo}CD11b^{hi}cells, and the Kupffer cells, considered as F4/80^{hi}CD11b^{lo}cells. **B.** Analysis of the expression of Arginase-1 (Arg1) considering events gated on the "Infiltrate" or "Kupffer cell" regions defined in panel A. **C.** Quantitative analysis of the populations indicated in panels A and B. Values are expressed as percentages. Asterisks represent relevant statistical differences between groups.

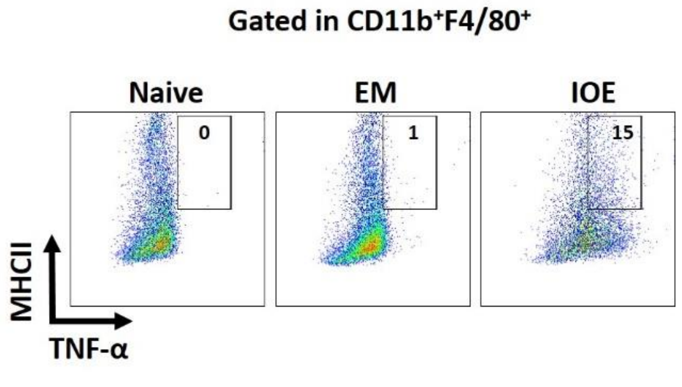

S4 Fig. Co-expression of MHC class II and TNF-α in BMM *in vitro*. Bone marrow-derived macrophages were infected of not with *Ehrlichia* (EM or IOE) and expression of polarization markers was assessed. Flow cytometry dot plot representations gated on live CD11b^{+}F4/80^{+} cells showing a region of co-expression of MHC class II and TNF-α. Numbers represent percentages of the population within the region.

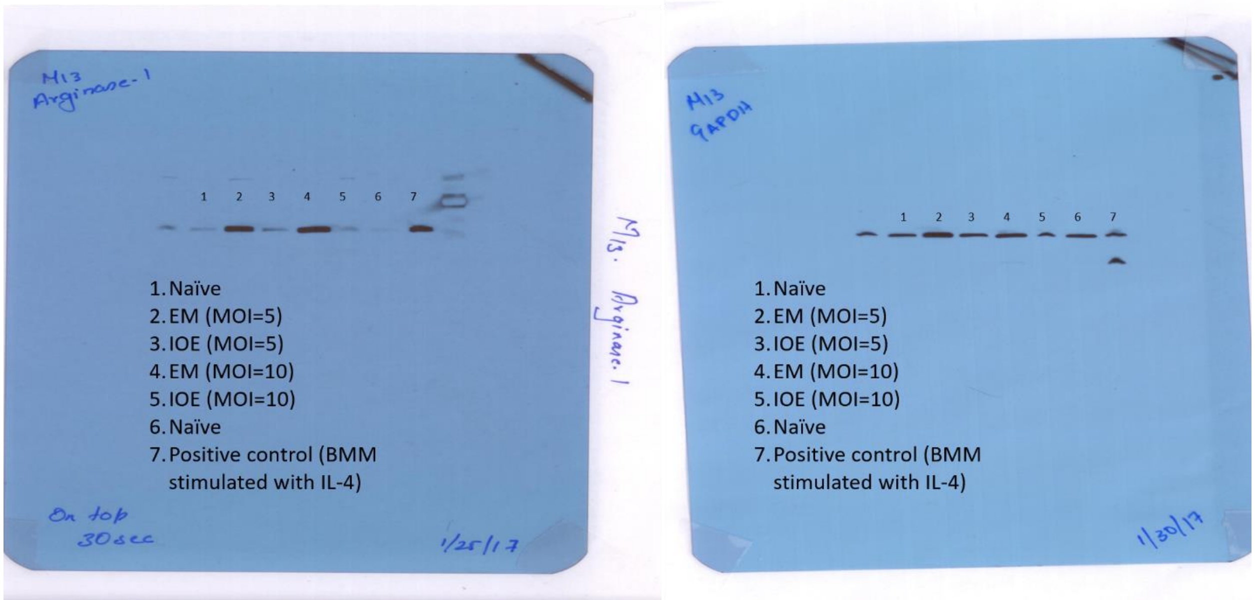

S5 Fig. Full-length blots of the analysis of arginase-1 expression (left) and GAPDH (right) as a load control. Data as reference to the Figure 4.

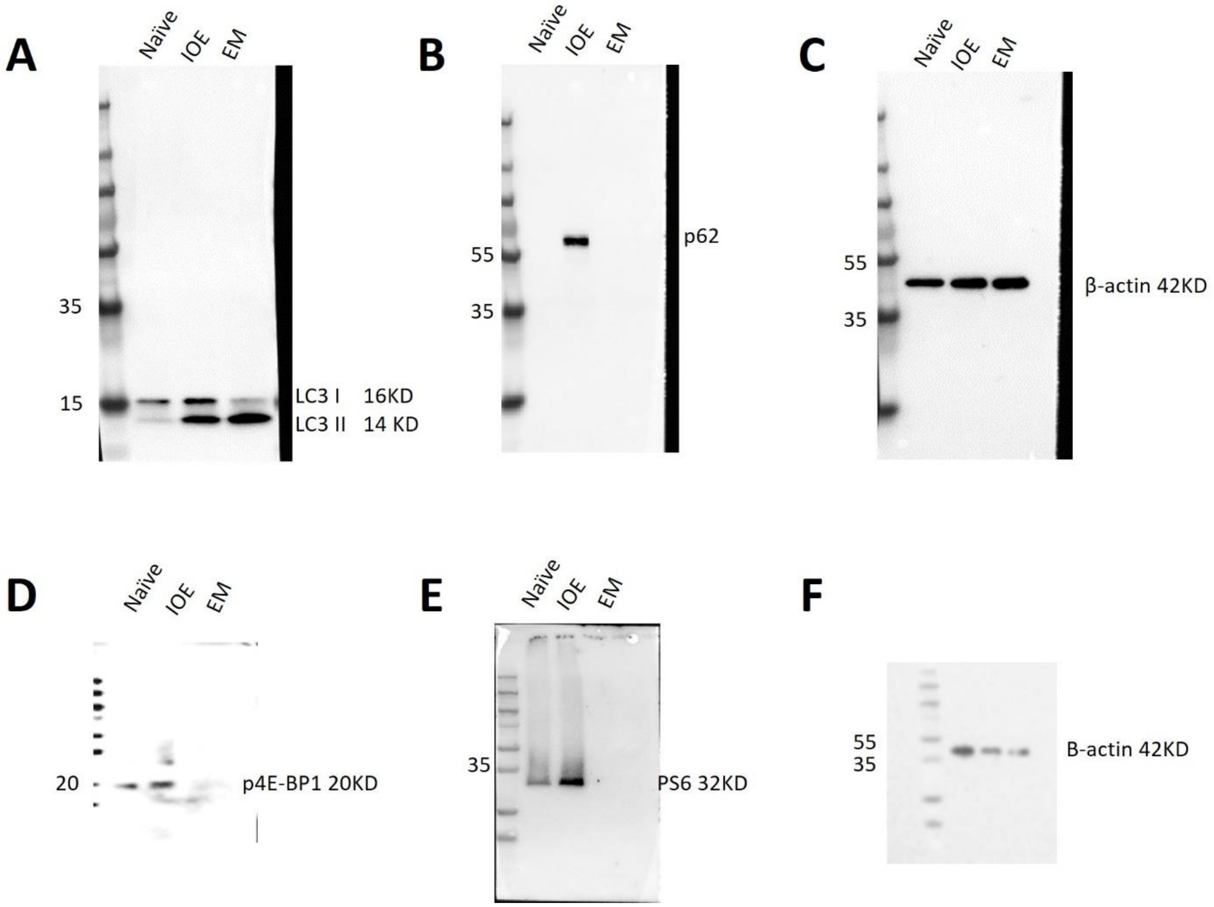

S6 Fig. Full-length membranes for the analysis of LC3 (A), p62 (B), p4EB-P1 (D) and pS6 (E). Expression of β-actin as a load control for LC3 and p62 is shown in panel C, and for p4EB-P1 and pS6 is shown in panel F. Data as reference to the Figure 5.

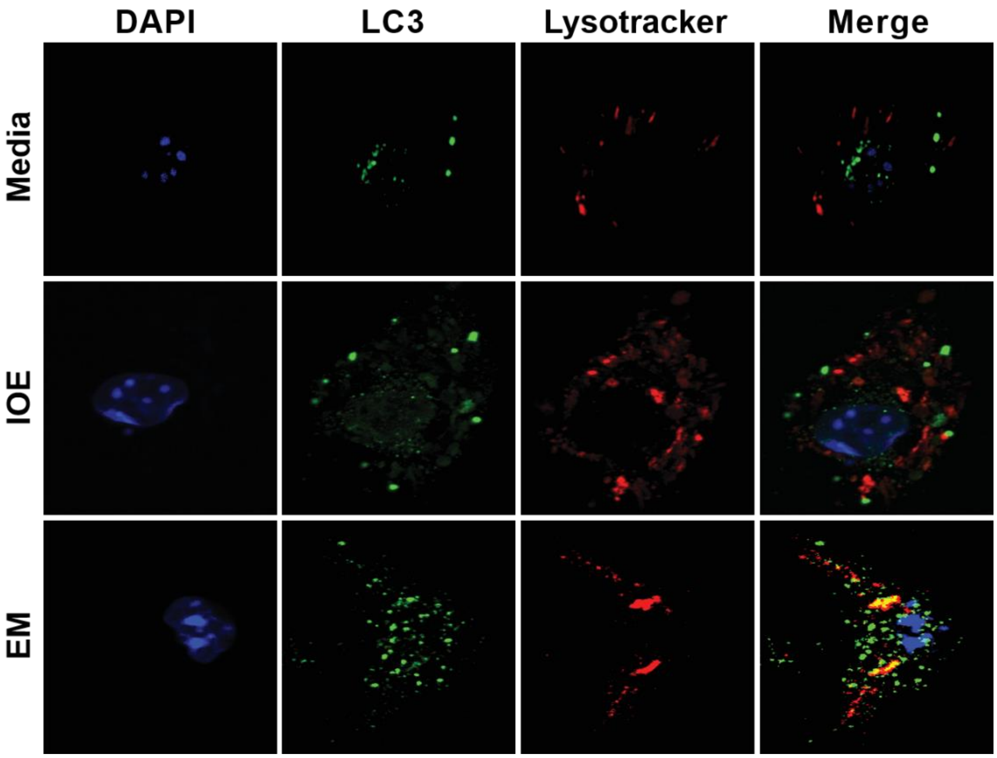

S7 Fig. Analysis of the autophagic flux in *Ehrlichia*-infected BMM by confocal microscopy. BMM were infected with E. *muris* (EM) or *Ixodes Ovatus Ehrlichia* (IOE) or left uninfected according to the experimental design. Cells were stained with anti-LC3 antibodies (green) and Lysotracker Red (staining lysosome) to visualize LC3 puncta and colocalization of LC3 with lysosome (yellow). DAPI was employed for nuclear stain, shown as blue. Representative confocal immunofluorescence showing higher autophagic induction (LC3 puncta) as well as autophagic flux (LC3-Lysotracker co-localization) in EM-infected BMM when compared to uninfected and IOE-infected BMM.
